# Anal lymphoma: a tumor with insufficient attention

**DOI:** 10.1007/s12672-023-00706-2

**Published:** 2023-06-05

**Authors:** Xibo Liu, Hongliang Chen

**Affiliations:** 1 Department of Pathology, Shaoxing People’s Hospital, No. 568, Zhongxing North Road, Shaoxing, 312000 Zhejiang Province China; 2 Department of Anorectal Surgery, Shaoxing People’s Hospital, No. 568, Zhongxing North Road, Shaoxing, 312000 Zhejiang Province China

**Keywords:** Anal canal, Lymphoma, Large B-Cell, Diffuse, Non-Hodgkin, Pathology

## Abstract

**Background:**

Anal lymphomas are extremely rare. There are no relevant descriptions in professional books, and there are only a few case reports in the literature. Here, we report a new case and review the literature to summarize the clinical and pathological features of anal lymphoma.

**Methods:**

We described a case of anal lymphoma confirmed by pathological diagnosis, then searched the PubMed database, and finally selected 12 reported cases to be included in the study. We described the clinical and pathological characteristics of the patients.

**Results:**

Thirteen patients with anal lymphoma were confirmed. Seven men and six women with a median age of 50. There were four cases of HIV- and EBV-infected patients. The size of the tumor was 1–13 cm, all of which were diagnosed as B-cell lymphoma, and 61.5% were diffuse large B-cell lymphomas. Among the 13 patients, eight received chemotherapy or immunochemotherapy, two received radiotherapy, one received chemotherapy combined with radiotherapy, one received surgery, and one gave up treatment. Three patients died, and only 2 of 10 surviving patients had complete remission.

**Conclusion:**

Anal lymphoma is extremely rare. Patients with persistent abscess complicated with HIV or EBV infection should undergo pathological biopsy to exclude anal lymphoma.

## Introduction

Lymphoma is a type of hematolymphoid tumor that is divided into Hodgkin and non-Hodgkin lymphomas based on the presence or absence of Reed-Sternberg cells, each of which has multiple subtypes [1]. Non-Hodgkin lymphoma is a neoplasm of lymphoid tissues originating from B cell precursors, mature B cells, T cell precursors, and mature T cells [2]. Lymphoma in the digestive system can be either a primary disease or part of systemic involvement. Primary gastrointestinal lymphomas are uncommon, accounting for approximately 10–15% of all non-Hodgkin lymphomas [3]. In the World Health Organization (WHO) Digestive System Tumor (5th Edition) [4], the anal canal is not mentioned in the location of lymphoma, which also indicates that the incidence rate of anal lymphoma is very low. Clinicopathological features of anal lymphoma are rarely described because lymphoma of the anal canal is extremely rare, with only a few reported cases in the literature since the first case reported by Steele in 1985 [5].

We encountered a case of anal lymphoma in our practical work, and then searched the English literature and found that a total of 12 cases were reported previously. Therefore, we summarized the clinical and pathological features of all 13 cases of anal lymphoma to provide a more detailed summary of the disease.

## Methods

### Case presentation

A 67-year-old man presented with symptoms of recurrent prolapse of the anal mass after defecation for more than 2 years ago. The mass gradually increased in size, and occasionally a small amount of blood dripped from the anus after defecation, accompanied by anal distention and discomfort. These symptoms recurred repeatedly and then gradually increased after exertion. The patient had no symptoms of anal pain, fecal incontinence, abdominal pain, diarrhea, constipation, or perianal discharge, and no B-symptoms. He had undergone hemorrhoidectomy 4 years previously. His uncle had a history of bladder cancer. No superficial lymphadenopathy, hepatomegaly, or splenomegaly was found on the physical examination. Digital anal examination revealed a skin-tag-like mass on the left anterior side of the anus, and mucosal congestion and swelling on the corresponding dentate line. No mass was found in the lower rectum. The serum lactate dehydrogenase level was 290.5 U/L. The HIV status of the patient was negative. No abnormalities were found in the routine blood and urine analyses. Ultrasonography revealed fatty liver and hepatic cysts with no gallbladder, pancreas, or splenic abnormalities. Radiographic examination revealed no abnormalities in either lung. The Zubrod-ECOG-WHO score was 1. The patient was diagnosed with mixed hemorrhoids and underwent mixed hemorrhoidectomy and internal hemorrhoid ligation under sacral anesthesia. The pathological diagnosis was anal DLBCL, and the patient refused further treatment at our hospital and went to a university hospital for immunochemotherapy (RCHOP plus lenalidomide) and radiotherapy.

### Retrieving cases

**Fig. 1:**
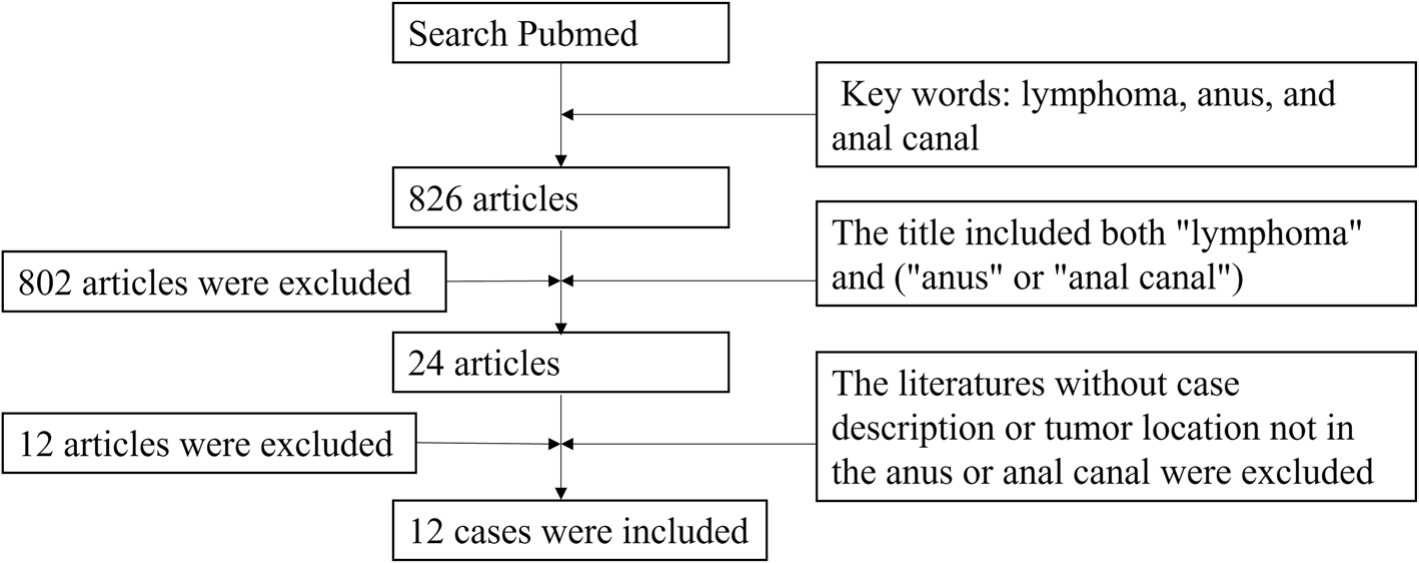
The criteria and process for the inclusion of research objects

We searched PubMed (https://pubmed.ncbi.nlm.nih.gov/) from 1950 to 2022 to identify the references for this article. We found 826 records by searching for the terms of lymphoma, anus, and anal canal. After browsing titles, 24 articles related to the research topic were selected. We excluded articles with no specific case description, tumor location not in the anus or anal canal, or no pathological diagnosis of non-Hodgkin lymphoma. Finally, 12 case studies were selected for review [1, 5–15], as shown in Fig. 1.

## Results

### Pathological examination

**Fig. 2:**
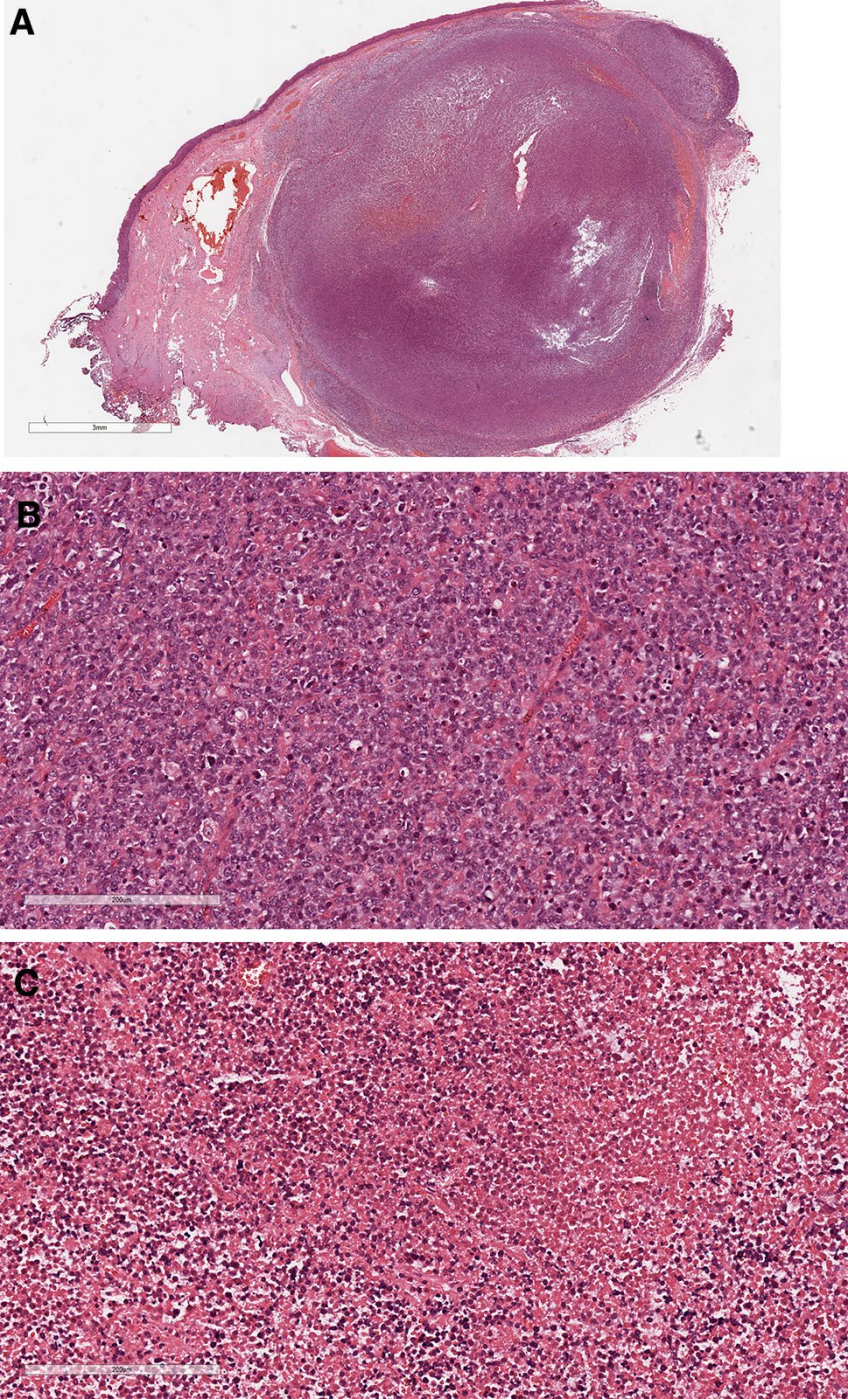
Histopathological images. **A** The mass was in the submucosal anal canal and formed a nodule with infiltrative growth accompanied by the formation of a microscopic satellite nodule. Scale bar = 3 mm. **B **The tumor cells were uniform in shape, medium to large, and diffusely distributed. Scale bar = 200 microns. **C** Local coagulative necrosis can be seen in the tumor tissue. Scale bar = 200 microns

**Fig. 3:**
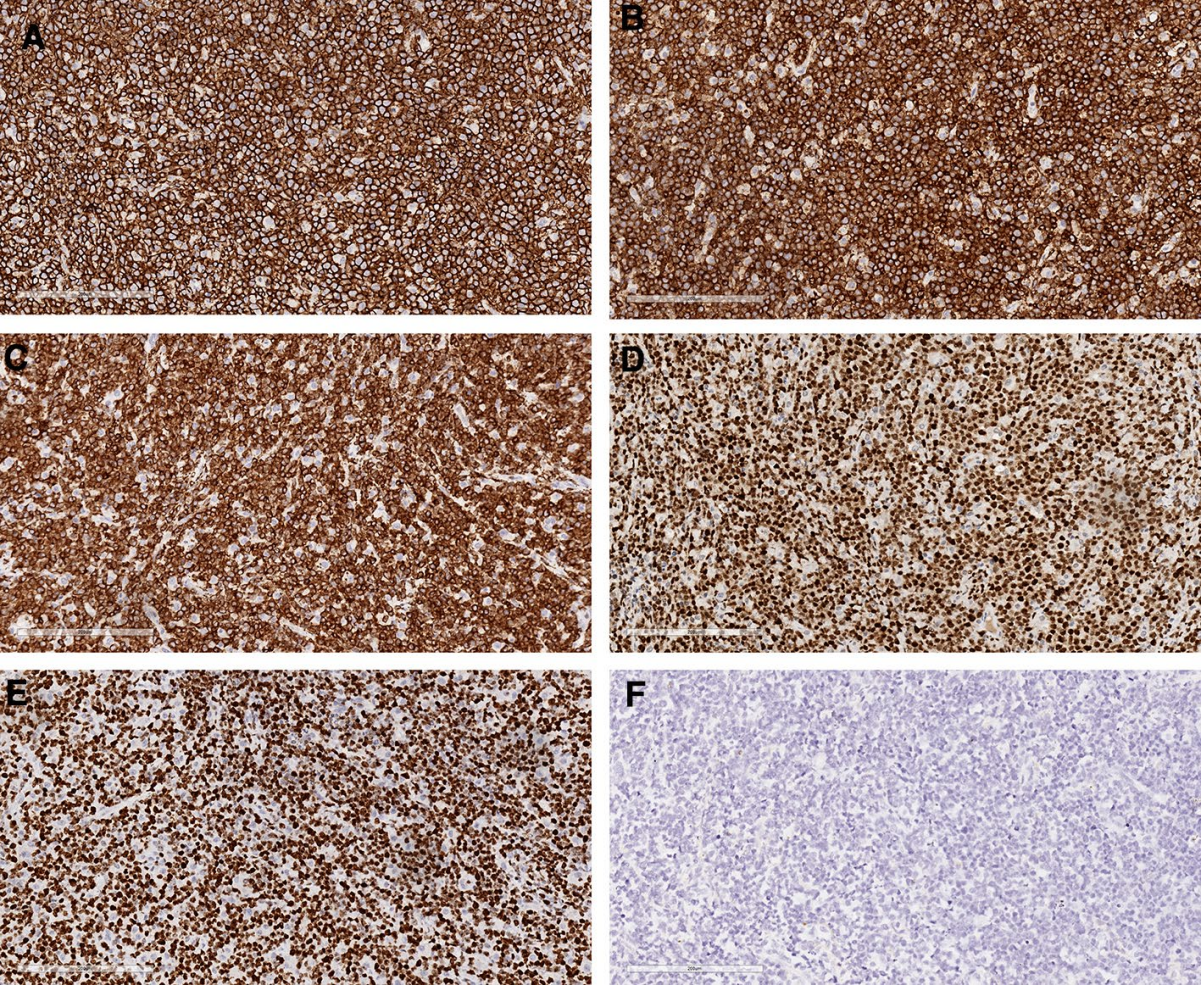
Immunohistochemical staining revealed positivity for CD20 (**A**), CD79α (**B**), bcl-2 (**C**), MUM-1 (**D**) and a high proliferative index for Ki-67 (**E**). EBER in situ hybridization was negative (**F**). Scale bar = 200 microns

Gross examination revealed a piece of skin-like tissue, 2 × 2 × 1 cm in size, and a gray-white nodule, 1 cm in diameter, was observed on the cut surface. Microscopic examination revealed a mass in the submucosal anal canal, forming a nodule with infiltrative growth accompanied by a microwave satellite nodule (Fig. 2A). The tumor cells were uniform in shape, medium to large, and diffusely distributed (Fig. 2B). Some tumor cells were necrotic, and an outline of the tumor cells was observed in the necrotic foci (Fig. 2C). Immunohistochemistry showed positivity for CD20 (Fig. 3A), CD79A (Fig. 3B), Bcl-2 (Fig. 3C) and MUM-1 (Fig. 3D) and negativity for CKpan, EMA, CD3, CD10, Bcl-6, CD21, CD45RO, S-100, HMB-45, Melan-A, CgA, Syn, and CD56. The Ki-67 proliferation index of tumor cells was as high as 95% (Fig. 3E). An in situ hybridization assay showed that the tumor cells were Epstein-Barr virus (EBV) negative (Fig. 3F). The pathological diagnosis was diffuse large B-cell lymphoma of non-germinal center origin.

### Imaging examination

**Fig. 4:**
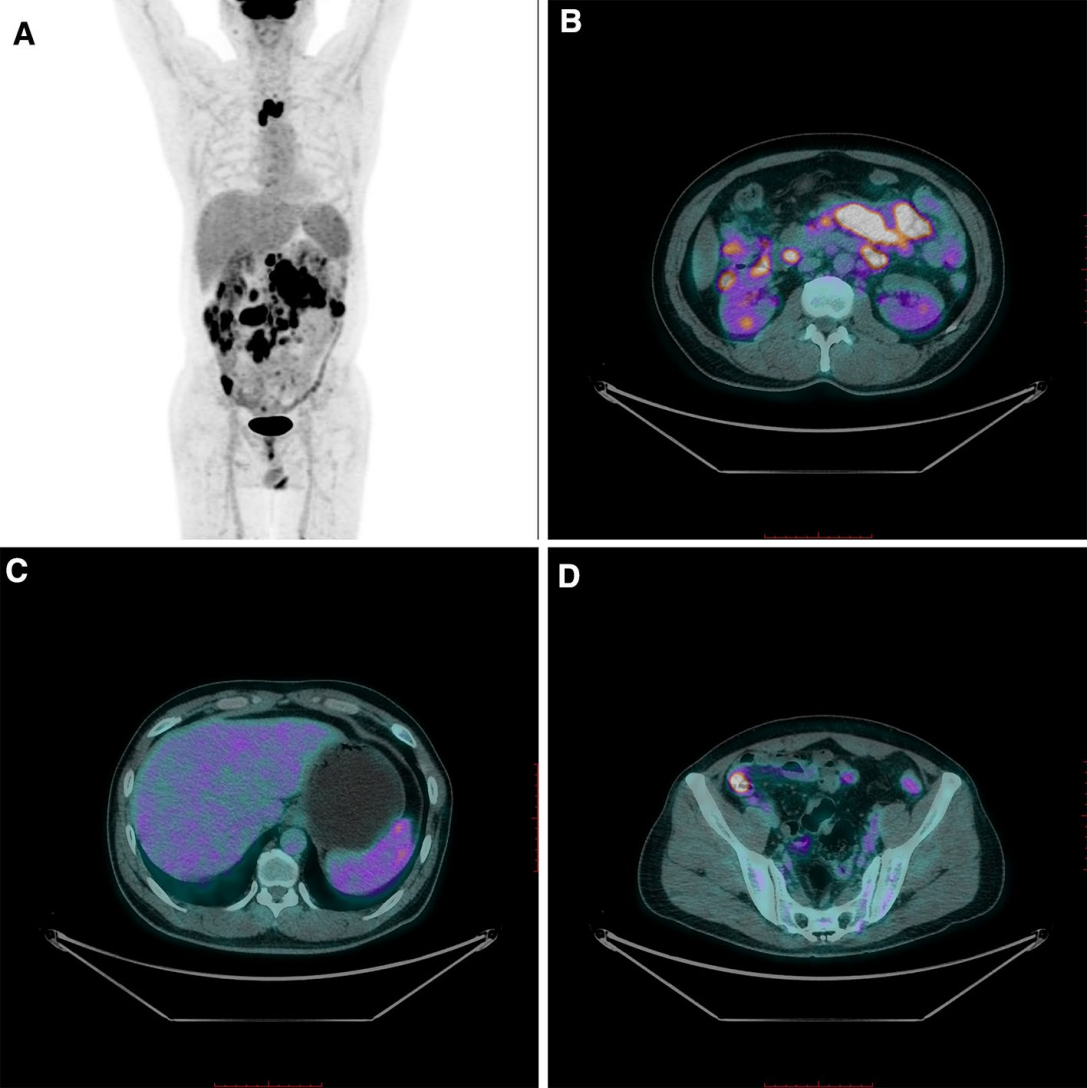
PET-CT examination revealed multiple enlarged lymph nodes with abnormally increased FDG metabolism (**A**, **B**). Diffuse increase in FDG metabolism in the spleen (**C**). Multiple patches of increased FDG metabolism in the ascending and descending colon (**D**)

Subsequent fluorodeoxyglucose-positron emission tomography (FDG-PET) revealed multiple enlarged lymph nodes in the superior anterior mediastinum and para-aortic, retroperitoneal, mesenteric, and bilateral paracolic grooves with abnormally increased FDG metabolism; some lymph nodes were fused (Fig. 4A and B). FDG metabolism in the spleen was diffusely increased, and FDG metabolism in the ascending and descending colons was increased in the patches (Fig. 4 C, D).

### Follow-up

The patient experienced disease progression during the RCHOP + lenalidomide treatment. One and a half months after discharge, the patient was readmitted due to hematochezia after chemotherapy. Laboratory tests revealed fecal red blood cell count of 25–35 / HP, fecal occult blood positivity. CT chest scan revealed intrahepatic low-density lesions. The patients were administered omeprazole acid suppression, sandostatin, carbazochrome sodium sulfonate hemostasis, teprenone to protect gastric mucosa, and compound amino acid support nutrition therapy. After 5 months, the patient developed anorexia and leukopenia and was treated with human granulocyte-stimulating factor. After 8 months, the patient had completed six courses of lymphoma chemotherapy (RCHOP + lenalidomide) when he visited the outpatient clinic for reexamination, and ultrasound examination revealed enlarged para-aortic lymph nodes. After 10 months, the patient visited our outpatient clinic with abdominal pain. The numerical rating scales (NRS) score was 4. The patient was administered tramadol hydrochloride sustained release tablets orally to relieve pain. Subsequently, the patient underwent positron emission tomography-CTP again. The results showed multiple enlarged lymph nodes in the systemic lymph system (splenic hilum, para-aortic region, retroperitoneal region, mesenteric region, right paracolon groove, bilateral pelvic wall, vesicorectal lacuna, right cardiac, and phrenic angle region) with abnormal increases in FDG metabolism and partial fusion. Multiple nodular FDG metabolic foci were observed in the liver capsule. The wall of the small intestine of group 6 and the sigmoid colon were significantly thickened with increased FDG metabolism. After 11 months, the patient still had pain symptoms, with an NRS score of 3, and continued to receive oral tramadol sustained-release tablets. Although the patient wanted further CAR-T treatment, the patient died of the disease one year after discharge.3.4 Clinical features.

The clinical characteristics of the 13 patients with anal non-Hodgkin lymphoma are shouwn in Table 1. DLBCL accounted for 61.5% of all cases. There was no significant sex difference among the 13 cases of anal or anal canal lymphoma; the male-to-female ratio was 1.17:1. The median age of the patients was 50 years, mean age was 56.6 years, andage at onset ranged from 38 to 89 years. The shortest time for symptom attack or tumor detection was two weeks, and the longest time was two years. The main clinical symptoms or complaints were pain (61.5%), bleeding (46.2%), mass prolapse (30.8%), abscess (38.5%), fecal incontinence (23.1%), discharge (15.4%), and weight loss (7.7%) %). Ulcers developed in five patients (38.5%). The tumor size ranged from 1 to 13 cm, with an average of 7.2 cm. Nine patients were tested for HIV, four of whom tested positive. Seven patients were tested for EBV, four of whom were positive.

**Table 1:** Clinicopathological features of patients with anal non-Hodgkin lymphoma

Author	Year	Gender	Age	History	Symptom	HIV	EBV	Pathology	Treatment	Outcome	Follow-up
Steele [5]	1985	F	89	Gastric ulceration	Pain, discharge	NR	NR	Centroblastic lymphoma	Radiotherapy	Alive with no evidence of disease	3 months
Porter [6]	1994	F	64	Non-Hodgkin’s lymphoma of right submandibular lymph node, Intraductal carcinoma of the breast	Pain, tenderness	Negative	NR	Diffuse large cell lymphoma	Radiotherapy	Alive with no evidence of disease	3 months
Smith [7]	1999	M	67	NR	Enlarging and ulcerated mass	Negative	NR	Large cell lymphoma, B cell type, immunoblastic	Chemotherapy	Alive with no evidence of disease	6 years
Ganeshan [8]	2002	M	66	NR	Perianal abscess	NR	NR	DLBCL	Chemotherapy	Alive with no evidence of disease	NR
Freudenberg [9]	2005	M	38	NR	Fecal incontinence, pain, weight loss, rapidly growing anal tumor	Positive	Positive*	BL	Abdominoperineal resection	Dead of disease	8 weeks
Lim [10]	2009	F	47	Tuberculosis, AIDS	Pain, bleeding, discharge	Positive	Positive*	PBL	Chemotherapy	NR	NR
Siddique [11]	2010	F	83	NR	Bleeding, prolapse, fecal incontinence, weight loss	NR	Positive*	DLBCL	None	NR	NR
Chagas [12]	2014	F	46	NR	Fecal incontinence	Positive	Negative+	PBL	Chemotherapy	Dead of other causes	In treatment
Jayasekera [1]	2014	M	63	No history of immune-compromised disease	Pain, fecal incontinence	NR	Positive*	DLBCL	Immunochemotherapy	NR	NR
Diamantopoulos [13]	2019	M	47	Unremarkable	Blooding, constipation, pain	Negative	NR	MALT	Immunochemotherapy	Alive with no evidence of disease	7 years
Kent [14]	2021	F	42	NR	Pain	Negative	Negative#	DLBCL	Immunochemotherapy	Alive with no evidence of disease	3 years
Tajudeen [15]	2021	M	40	NR	Bleeding, pain, spurious diarrhea	Positive	NR	PBL	Chemotherapy and radiotherapy	Alive with no evidence of disease	Under follow-up
Liu	Now	M	53	Mixed hemorrhoids	Prolapse, bleeding	Negative	Negative*	DLBCL	Immunochemotherapy	Dead of disease	one year

*M *male, *F* female, *NR* no narrative, *AIDS* acquired immunodeficiency syndrome, *HIV* human immunodeficiency virus, *EBV* Epstein-Barr virus, *BL* Burkitt lymphoma, *DLBCL* diffuse large B-cell lymphoma, *MALT* extranodal marginal zone lymphoma of mucosa-associated lymphoid tissue, *PBL* plasmablastic lymphoma.

*In situ hybridization of fluorescein

+Polymerase chain reaction

#Immunohistochemical staining

For consistency of terminology, the conditions previously described as immunoblastic lymphomas, centroblastomas, and diffuse large cell lymphomas were all classified as diffuse large B-cell lymphomas. All patients in this group were pathologically diagnosed with non-Hodgkin lymphoma, all of whom presented with B-cell lymphomas. In this group, there were 8 cases (61.5%) of diffuse large B-cell lymphoma, 3 cases (23.1%) of plasmablastic lymphoma, 1 case of Burkitt lymphoma, and 1 case of mucosa-associated lymphoma lymphoid tissue lymphoma (7.7% each).

Among the 13 patients, eight received chemotherapy or immunochemotherapy, two received radiotherapy, one received chemotherapy combined with radiotherapy, one received surgery, and one gave up treatment. The follow-up results showed that 3 patients died, and only 2 of 10 surviving patients had complete remission.

## Discussion

Non-Hodgkin’s lymphomas are a heterogeneous group of cancers of the immune system that can involve any organ in the body, have a broad range of presentations that span the spectrum of indolent to very aggressive clinical behavior, and can be observed by physicians from most specialties [16]. Although extranodal lymphomas account for one-third of all non-Hodgkin lymphomas, the gastrointestinal tract is the most common extranodal site. Primary colorectal lymphomas are extremely rare, accounting for less than 1% of all colorectal malignancies. Primary lymphoma of the anal canal accounts for approximately 0.1% of all anal malignancies [14]. Most non-Hodgkin lymphomas involving the gastrointestinal tract belong to the B-cell lineage, with diffuse large B-cell lymphoma being the most common subtype regardless of location.

Anal lymphomas often present as perianal abscesses, possibly due to infection of the anal glands or obstruction by tumor infiltration [5, 6]. Therefore, some surgeons believe that all anorectal abscesses should be biopsied for pathological examinations [8]. An increasing number of cases of anorectal lymphoma associated with acquired immunodeficiency syndrome have been reported, most of which involve homosexual men [7]. People with AIDS have a 60-fold higher risk of developing non-Hodgkin lymphoma than the general population. [8] Therefore, non-Hodgkin lymphoma is the second most common malignancy in HIV-positive patients [10, 12]. When encountering a perianal abscess that cannot heal, it must be suspected. Good tissue biopsy and histological monitoring are critical in patients with unresolved perianal disease [1]. EBV is a herpes virus that affects 90% of the world’s population. T-cell immunity does not have any adverse effects on immunocompetent hosts; however, long-term viral carriage results in the appearance of many EBV-positive tumors [11].

Primary gastrointestinal lymphoma is defined by the following five components [17]: (1) no palpable superficial lymph nodes at presentation, (2) no mediastinal lymphadenopathy on chest radiography, (3) a normal range of white blood cell counts, including total number and classification, (4) only regional lymph nodes involved at the time of surgery, and (5) no liver or spleen disease. The patients in this group were treated for anal lymphoma as the main symptom, and some were found to have lymph node involvement in the diagnosis and treatment process. [12, 13] However, some reports did not provide the results of systemic lymph node examinations. Thus, cases with anal lymphoma as the first symptom or chief complaint may be primary anal lymphoma or represent a systemic disease.

In our group of cases, five patients underwent PET-CT examination; two had no lymph node uptake. In one of the three patients with abnormal fluorodeoxyglucose uptake in the lymph nodes on PET-CT, lymphoma was excluded by biopsy. Because our patient had a history of hemorrhoidectomy, we reviewed the previous pathology slides and confirmed no previous evidence of lymphoma.

Immunohistochemistry has several essential applications in lymphoma diagnosis, including the identification of the cell lineage and phase of maturation, detection of specific genetic alterations, visualization of the degree of cell proliferation, and identification of therapeutic targets [18]. Moreover, immunohistochemistry can be used for differential clinicopathological diagnoses. Anal lymphoma must be differentiated from the following diseases: poorly differentiated adenocarcinomas that can form nested or cord-like structures with intracellular or extracellular mucinous components and immunohistochemical expression of epithelial markers such as cytokeratin, EMA, and carcinoembryonic antigen (CEA).

Melanomas can also occur in the anus. The histological features were similar to those of cutaneous melanoma, with a borderline component adjacent to the infiltrating tumor. Diagnosis is challenging and requires immunohistochemical confirmation of tumor cells expressing S-100, HMB-45, and Melan A. The immunohistochemical results of our patient did not support a diagnosis of malignant melanoma [19].

Neuroendocrine carcinomas can occur anywhere in the body; however, neuroendocrine tumors in the anorectal region are extremely rare. Morphologically, poorly differentiated neuroendocrine carcinomas are mostly patchy or diffuse in structure, with irregular nuclei, few cytoplasmic granules, and incomplete or negligible expression of neuroendocrine markers. They exhibit immunohistochemical reactivity to neuroendocrine markers, such as chromogranin and synaptophysin. Small cell carcinomas of the anal canal may exhibit immunoreactivity for thyroid transcription factor-1 and CK [19].

The treatment of NHL varies greatly depending on tumor stage, grade, type of lymphoma, and various patient factors (e.g., symptoms, age, and performance status) [2]. Patients with localized large B-cell lymphoma typically receive a combination chemoimmunotherapy and field radiation therapy [20]. Although antibodies that bind to the CD20 antigen on B cells (G1 monoclonal antibody rituximab) are widely used clinically for the treatment of non-Hodgkin’s lymphoma [11], standard first-line immunochemotherapy regimens for DLBCL include rituximab plus chlorambucil, rituximab-bendamustine, rituximab, cyclophosphamide, doxorubicin, vincristine, and prednisone (R-CHOP) [13]. For localized disease, R-CHOP regimen can be curative for localized disease [14]. For patients with large masses, the addition of radiation therapy may improve outcomes [20]. Recent studies have shown that targeting the CD19 antigen on B cells with autologous chimeric antigen receptor (CAR) cells is highly effective in patients with relapsed and refractory DLBCL [21].

Seven patients were followed up, with the longest being 7 years. Two patients who died were treated with surgery and chemotherapy, respectively. Two of the ten surviving patients responded completely. Our patient underwent immunochemotherapy and radioactive particle therapy at another hospital after discharge. During the treatment period, the patient showed disease progression and new lesions appeared, ultimately leading to death.

In summary, because anal non-Hodgkin lymphoma is extremely rare, surgeons usually treat it as benign during the patient’s first visit. Caution should be exercised in patients presenting persistent with abscesses and HIV or EBV infection. A definitive diagnosis requires a biopsy and pathological examination. DLBCL is the primary pathological type of anal lymphoma. The treatment of anal non-Hodgkin lymphoma is similar to that of lymphomas at other sites. Owing to the lack of sufficient samples, the prognosis remains to be determined.

## Data Availability

The datasets generated during and/or analyzed during the current study are available from the corresponding author on reasonable request.

## Abbreviations

AIDS: Acquired immunodeficiency syndrome
HIV: Human immunodeficiency virus
EBV: Epstein-Barr virus
BL: Burkitt lymphoma
DLBCL: Diffuse large B-cell lymphoma
MALT: Extranodal marginal zone lymphoma of mucosa-associated lymphoid tissue
PBL: Plasmablastic lymphoma
FDG: Fluorodeoxyglucose
PET: Positron emission tomography
